# Pulmonary eosinophilia may indicate onset stage of allergic bronchopulmonary aspergillosis

**DOI:** 10.1186/s13223-021-00624-4

**Published:** 2021-11-18

**Authors:** Mari Miki, Yuko Ohara, Kazuyuki Tsujino, Takahiro Kawasaki, Tomoki Kuge, Yuji Yamamoto, Takanori Matsuki, Keisuke Miki, Hiroshi Kida

**Affiliations:** 1 Department of Respiratory Medicine, National Hospital Organization Osaka Toneyama Medical Center, Osaka, Japan; 2 Department of Internal Medicine, Tokushima Prefecture Naruto Hospital, 32 Kotani, Kurosaki, Muya-cho, Naruto, Tokushima 772-8503 Japan; 3 Department of Respiratory Medicine, Ikuwakai Memorial Hospital, Osaka, Japan

**Keywords:** *Aspergillus*, Eosinophilia, Eosinophilic pneumonia, Ground-glass opacities, Immunoglobulin E

## Abstract

**Background:**

Allergic bronchopulmonary aspergillosis (ABPA) and chronic eosinophilic pneumonia (CEP) both display peripheral eosinophilia as well as pulmonary infiltration, together described as pulmonary eosinophilia, and differentiation is sometimes problematic. This study therefore examined the distinctions between ABPA with and without CEP-like shadows.

**Methods:**

This retrospective cohort study from a single center included 25 outpatients (median age, 65 years) with ABPA diagnosed between April 2015 and March 2019, using criteria proposed by the International Society of Human and Animal Mycology (ISHAM), which focuses on positive specific IgE for *Aspergillus fumigatus*. Patients were assigned to either the eosinophilic pneumonia (EP) group or Non-EP group, defined according to findings on high-resolution computed tomography (HRCT). The EP group included patients with HRCT findings compatible with CEP; i.e., the presence of peripheral consolidation (p-consolidation) or ground-glass opacities (GGO), with no evidence of high-attenuation mucus. The Non-EP group comprised the remaining patients, who showed classical findings of ABPA such as mucoid impaction. Differences between the groups were analyzed.

**Results:**

Baseline characteristics, frequency of a history of CEP (EP, 50% vs. Non-EP, 26%) and tentative diagnosis of CEP before diagnosis of ABPA (67% vs. 16%) did not differ significantly between groups. Although elevated absolute eosinophil count and *Aspergillus*-specific immunoglobulin E titers did not differ significantly between groups, the Non-EP group showed a strong positive correlation between these values (R  = 0.7878, p  = 0.0003). The Non-EP group displayed significantly higher levels of the fungal marker beta-D glucan (median, 11.7 pg/ml; interquartile range, 6.7–18.4 pg/ml) than the EP group (median, 6.6 pg/ml; interquartile range, 5.2–9.3 pg/ml). Both groups exhibited frequent recurrence of shadows on X-rays but no cases in the EP group had progressed to the Non-EP group at the time of relapse.

**Conclusions:**

The ABPA subgroup with imaging findings resembling CEP experienced frequent recurrences, as in typical ABPA. In pulmonary eosinophilia, even if there are no shadows indicating apparent mucous change, the *Aspergillus*-specific immunoglobulin E level is important in obtaining an accurate diagnosis and in the selection of appropriate therapies for this type of ABPA.

## Background

Allergic bronchopulmonary aspergillosis (ABPA) is a pulmonary disorder caused by type 2 immune responses to *Aspergillus*. This pathology usually develops after the onset of bronchial asthma (BA) or cystic fibrosis [1] and prevalence rates of ABPA among adult asthmatic patients have been estimated as 2.5% in China and 7–22% in India [2]. Rates differ among countries but appear to be increasing around the world with the increase of BA [3].

Several diagnostic criteria for ABPA have been proposed, such as a history of BA or cystic fibrosis, characteristic radiographic pulmonary opacities including central dilatation of the bronchus, elevated immunoglobulin (Ig)E levels, and hypersensitivity reaction to *Aspergillus* including elevated IgE antibodies against *Aspergillus fumigatus* and/or IgG antibodies for *Aspergillus* [2, 4]. In some cases, however, onset is sudden or potentially before recognition of the precedent diseases, and the proposed diagnostic criteria have proved inadequate for keeping track of all patients with ABPA, especially in cases without apparent preceding BA [5, 6]. Furthermore, the detailed clinical and laboratory data characteristics of ABPA remain unclear even to pulmonologists, which might delay diagnosis and thus cause irreversible pulmonary damage [7].

This study retrospectively investigated the clinical characteristics of patients with ABPA and examined the obstacles to appropriate diagnosis. We found that in several cases, chronic eosinophilic pneumonia (CEP) had initially been the suspected diagnosis. Pulmonary eosinophilia is common to both CEP and ABPA, and is apparent radiographically as transient pulmonary shadows, in association with elevated blood eosinophil count [8]. Few studies have reported a connection between CEP and ABPA [8–11] and to the best of our knowledge, none has described the clinical characteristics of patients with ABPA who show CEP-like shadows on high-resolution computed tomography (HRCT). The aim of the present study was to clarify the clinical features and the significance of specific IgE antibodies against *A. fumigatus* between patients stratified into groups according to the presence or absence of CEP-like shadows on HRCT, to improve the accuracy of diagnosis of CEP-like ABPA.

## Methods

This retrospective, single-center study was approved by the institutional research ethics committee of National Hospital Organization Osaka Toneyama Medical Center, Japan (No. TNH-2019055). The study was conducted in accordance with the principles of the Declaration of Helsinki and the need to obtain written informed consent was waived due to the retrospective nature of the analysis. The medical records for outpatients diagnosed with BA based on the Global Initiative for Asthma (GINA) guidelines [12] between April 2015 and March 2019 were carefully investigated, and the selected patients were followed up for at least 1 year.

### Study patients

Patients were included in the study if they fulfilled the diagnostic criteria for ABPA proposed by the International Society of Human and Animal Mycology (ISHAM) [2], which included a history of BA or cystic fibrosis and both of the following: (1) positive immediate cutaneous hypersensitivity to *Aspergillus* antigen or specific IgE to *A. fumigatus*, and (2) elevated total IgE level in serum  > 1000 IU/ml. In addition, the ISHAM criteria require patients to have at least two of the three following features for diagnosis: (1) presence of precipitating or IgG antibodies against *A. fumigatus* in serum; (2) pulmonary opacities consistent with ABPA on radiographic imaging; and (3) total eosinophil count  > 500 cells/µl in steroid-naïve patients (may be historical). Patients with any of the following were excluded: (1) diagnosis of aspergilloma, chronic pulmonary aspergillosis, or invasive pulmonary aspergillosis; (2) presence of broad shadows above one lobe due to other respiratory diseases such as sequelae of pulmonary tuberculosis (TB); or (3) positive culture of *Schizophyllum* from sputum or bronchial wash.

### Data collection and study procedure

Data of patients of age, sex, medical history (with a focus on childhood asthma and pulmonary diseases, especially CEP), age at ABPA onset, and underlying diseases were collected from each patient’s medical record. Data of total eosinophil count, serum IgE (total and *A. fumigatus*-specific), *Aspergillus*-specific precipitating antibody confirmed by Ouchterlony double immunodiffusion testing or IgG evaluated by the complement fixation method, spirometry, chest radiographs, and HRCT at onset of ABPA were also collected. HRCT of the chest was performed using a 64-row, multiple-detector row CT scanner (SOMATOM Definition AS  +, Siemens, Munich Germany). Image analysis was performed by two chest physicians under the direction of a radiologist. Central bronchiectasis was defined as bronchiectasis present in the medial two-thirds of lung parenchyma [13], and mucoid impaction and lymphadenopathy were evaluated. High-attenuation mucus (HAM) was defined as mucus showing a higher CT attenuation value than paraspinal skeletal muscle [7, 14]. In this study, we used the mean CT attenuation values in areas of 10 mm^2^ to evaluate small regions of HAM. Also evaluated were peripheral consolidation (p-consolidation), ground-glass opacities (GGO), fibrosis, and tree-in-bud appearance indicative of bronchiolitis characterized by centrilobular nodules and branching linear or nodular areas.

### Definition of the eosinophilic pneumonia (EP) and Non-EP groups

**Fig. 1:**
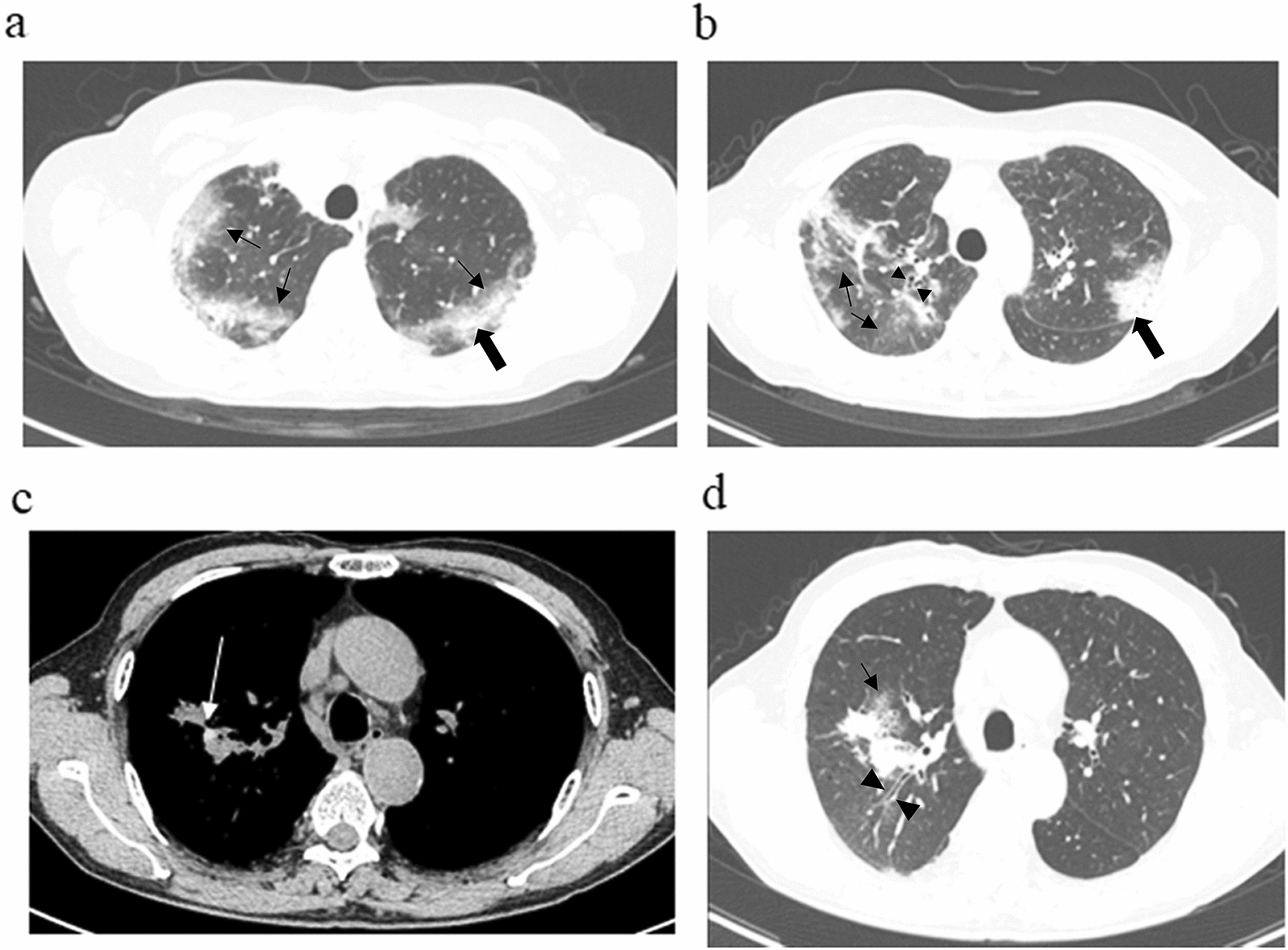
Representative CT images of the EP and non-EP groups. **a** EP group, Case 1: pre-treatment HRCT shows infiltrates with the appearance of ground-glass opacities (small arrows) and dense consolidation (large arrow) predominantly in the peripheral upper lobes. **b** EP-group, Case 2: HRCT scan shows infiltrates with the appearance of ground-glass opacities (small arrows) and consolidation (large arrow) with bronchial wall thickening (small arrowheads)**,** predominantly in the peripheral upper lobes. Non-EP group, Case 3 (**c** mediastinal window; **d** lung window): HRCT scans show patchy ground-glass opacities (small arrow) and slight HAM impaction (white arrow in **c**) with bronchiectasis (arrowheads) and lymph node swelling

Air-space consolidation with p-consolidation and GGO on HRCT are typical findings of CEP [15, 16]. Therefore, we defined the EP group as patients who showed peripheral eosinophilia and findings compatible with CEP on HRCT; specifically, an absence of HAM and the presence of p-consolidation or GGO on CT. All remaining patients were allocated to the Non-EP group; these patients showed the classical ABPA findings of mucoid impaction or HAM on HRCT. Central bronchiectasis could be seen in both groups, but the peripheral lung findings took priority over ectasis on HRCT in the group allocations (Fig. 1).

### Statistical analysis

All statistical analyses were performed using JMP version 11 software (SAS Institute, Cary, NC). Continuous variables are expressed as median and interquartile range (IQR) and categorical data are presented as number and percentage. Patient groups were compared using the Mann–Whitney U test for continuous variables. Differences between categorical variables were analyzed using the χ^2^ test or Fisher’s exact probability test. Statistical significance was established at the level of p  < 0.05.

## Results

A total of 32 patients with physician-diagnosed ABPA were identified from medical records during the study period. Among these, 7 patients were excluded from analysis for the reasons of pulmonary complications making image evaluation difficult in 5 patients (sequelae of pulmonary tuberculosis, n  = 1; sequelae of pulmonary tuberculosis and aspergilloma, n  = 2; simple aspergilloma, n  = 2); and unfulfilled diagnostic criteria of ISHAM despite HAM on CT (no data on *Aspergillus*-specific IgE, n  = 1; low level of IgE, n  = 1). A total of 25 patients met the ISHAM criteria for ABPA and were enrolled in the study (Table 1). Median patient age was 65 years (IQR, 56–77 years) and female patients accounted for 60% of study participants. Smoking history was observed in 4 patients (16%) and a history of infantile asthma or CEP were present in a substantial minority of patients (32% and 28%, respectively), with some overlap. Tentative diagnoses from other hospitals or clinics included ABPA (n  = 6, 24%), CEP (n  = 7, 28%), and mycobacteriosis [TB, n  = 4; non-tuberculous mycobacteria (NTM), n  = 2] even if CT was obtained.

**Table 1:** Baseline characteristics of 25 patients with ABPA

Characteristic	All cases (n = 25)	EP group (n = 6)	Non-EP group (n = 19)	p value
	No. (%) or median (IQR)			
Age at onset of ABPA (y), median (IQR)	65 (56–71)	67 (61–73)	65 (55–71)	ns
Sex, female	15 (60%)	4 (67%)	11 (58%)	ns
Bronchial asthma				
Age at onset (y), median (IQR)	50.0 (36.0–64.0)	49.0 (31.5–50.0)	55.0 (38.0–66.5)	ns
Duration (y) between onset of asthma and ABPA, median (IQR)	3.0 (0.4–16.0)	18.0 (4.2–24.5)	2.0 (0.5–8.0)	ns
History				
Smoking status, never/former/current	21 (84%)/3 (12%)/1 (4%)	5 (83%)/1 (17%)/0 (0%)	16 (84%)/2 (11%)/1 (5%)	ns
Infantile asthma	8 (32%)	2 (33%)	6 (31.6%)	ns
CEP	8 (32%)	3 (50%)	5 (26%)	ns
Other respiratory disease	4 (16%)	0	4	
COPD	2 (8%)	0	2	ns
Sequelae of pulmonary tuberculosis	1 (4%)	0	1	ns
NTM	1 (4%)	0	1	ns
Tentative diagnosis				
ABPA	6 (24%)	0 (0%)	6 (32%)	ns
CEP	7 (28%)	4 (67%)	3 (16%)	ns
TB	4 (16%)	0 (0%)	4 (21%)	ns
NTM	2 (8%)	1 (17%)	1 (5%)	ns
Pneumonia	2 (8%)	1 (17%)	1 (5%)	ns
LC	2 (8%)	0 (0%)	2 (11%)	ns
Bronchiectasis	1 (4%)	0 (0%)	1 (5%)	ns
Middle lobe syndrome	1 (4%)	0 (0%)	1 (5%)	ns
Laboratory data at diagnosis				
Absolute eosinophil count (cells/μl), median (IQR)	1540 (880–2330)	2385 (1663–2733)	1240 (560–1700)	ns
IgE (IU/ml), median (IQR)	2802 (1330–5182)	3010 (1131–4227)	2802 (1809–5182)	ns
*Aspergillus*-specific IgE (IU/ml), median (IQR)	20.7 (7.0–33.4)	25.8 (8.6–31.5)	20.6 (1.7–33.7)	ns
*Aspergillus*-specific precipitating antibodies or IgG	16 (64%)	6 (100%)	10 (53%)	p = 0.0664
Fungal culture, beta-D glucan				
Sputum or bronchial wash positive	11 (44%)	1 (17%)	10 (53%)	ns
Sputum; wash; sputum and wash	4; 3; 4	1; 0; 0	3; 4; 3	
*Aspergillus fumigatus*	8 (32%)	0 (0%)	8 (42%)	ns
*Aspergillus niger*	2 (8%)	1 (17%)	1 (5%)	ns
*Aspergillus fumigatus* and *niger*	1 (4%)	0 (0%)	1 (5%)	ns
beta-D glucan (pg/ml)	11.1 (5.3–16.6)	6.6 (5.2–9.3)	11.7 (6.7–18.4)	p = 0.0398
Chest CT findings				
Central bronchiectasis	22 (88%)	4 (67%)	18 (95%)	ns
Mucoid impaction	19 (76%)	0 (0%)	18 (95%)	p = 0.0006
HAM	13 (52%)	0 (0%)	12 (63%)	p = 0.0052
Tree in bud	12 (48%)	3 (50%)	9 (47%)	ns
Enlarged lymph nodes	12 (48%)	3 (50%)	9 (47%)	ns
Fibrosis	0 (0%)	0 (0%)	0 (0%)	ns
Peripheral consolidation	16 (64%)	6 (100%)	10 (53%)	p = 0.0571
Ground grass opacities	16 (64%)	6 100%)	10 (53%)	p = 0.0571
Pulmonary function test				
FEV_1_, L, median (IQR)	1.74 (1.46–2.25)	1.60 (1.27–2.35)	1.82 (1.48–2.25)	ns
FEV_1_, %, predicted, median (IQR)	82.6 (72.0–91.8)	73.3 (65.9–81.1)	85.8 (74.7–89.4)	ns
FEV_1_/FVC, %, median (IQR)	72.7 (62.0–91.8)	71.4 (62.2–77.2)	72.7 (62.4–77.3)	ns
Recurrence	21 (84%)	4 (67%)	15 (78%)	ns
Duration (months) before recurrence	21.5 (14.8–29.0)	22.5 (20.5–22.9)	20.5 (13.8–34.8)	ns
Recurrence rate	84%	67%	89%	ns

Data are expressed as median and interquartile range (IQR) and categorical data are presented as number and percentage

p values between the EP group and Non-EP group are shown

ns, not significant

All patients showed peripheral eosinophilia (median, 1540 cells/μl), elevated level of IgE (median, 2802 IU/ml) and positive reactions for *Aspergillus*-specific IgE (median, 20.7 IU/ml; IQR, 0.9–81.2 IU/ml). In addition, 64% of patients had a positive reaction for *Aspergillus*-specific antibodies, mainly representing IgG. Sputum culture was obtained in all 25 patients and cultures of bronchial wash obtained by bronchoscopy were available in 20 patients. *Aspergillus* spp. were identified in 44% of all patients: in sputum in 32%, bronchial wash in 35%, and both in 16%. *A. fumigatus* alone was cultured most commonly, in 32% of patients, followed by *A. niger* in 8%, and both *A. fumigatus* and *A. niger* in 4%.

The most common HRCT findings were central bronchiectasis (88%) and mucoid impaction (77%), with HAM found in 13 cases (52%). FEV_1_/FVC% was  < 70% in 22% and most patients showed no evidence of any obstructive disorder.

### Comparison between the EP and Non-EP groups

The baseline characteristics of the two groups (Table 1) did not differ significantly in terms of age at onset of ABPA, ratio of females, or duration between onset of asthma and onset of ABPA. A history of CEP was present in 50% of the EP group and 26% of the Non-EP group. In patients who had been diagnosed with CEP in the past, *Aspergillus*-specific IgE had not been checked at the time of diagnosis of CEP. Among these patients, one patient in the EP group had a positive sputum culture of *A. fumigatus* and branched fungus was detected in another patient by microscopy of sputum, and all patients had the similar CT patterns at the time of diagnosis of ABPA. With respect to tentative diagnoses, CEP was diagnosed in 67% of the EP group and in 16% of the Non-EP group, whereas ABPA was present in 32% of the Non-EP group and completely absent in the EP group. Median eosinophil count and IgE and *Aspergillus*-specific IgE titers at diagnosis tended to be higher in the EP group and positive reaction rates for *Aspergillus*-specific antibody also tended to be higher in the EP group (100% vs. 53%, p  = 0.0664). Levels of beta-D-glucan, a fungal marker, were significantly higher in the Non-EP group (median, 11.7 pg/ml; IQR, 6.7–18.4 pg/ml) than in the EP group (median, 6.6 pg/ml; IQR, 5.2–9.3 pg/ml), but there was no significant difference in positive culture rates for fungi between the groups. These results indicate that the Non-EP group had a stronger relationship with fungal infection than did the EP group. On HRCT, the Non-EP group showed significantly higher rates of mucous changes in the bronchus, such as mucoid impaction (89% vs. 33%, p  = 0.0006) and HAM (63% vs. 0%, p  = 0.0052), compared with the EP group. In contrast, p-consolidation and GGO in HRCT were present in all patients in the EP group and in only 53% of the Non-EP group (p  = 0.0571). Central bronchiectasis, lymph node swelling, tree-in-bud sign, and GGO were found at similar frequencies in both groups. On chest CT, the EP group had imaging findings of peripheral-dominant lung changes, whereas the Non-EP group showed changes mainly in bronchial regions.

Most patients in the EP group had received oral corticosteroids (5/6, 83%) and only 1 patient had received anti-fungal therapy (for positive *A. niger* culture), whereas most patients in the Non-EP group had received anti-fungal therapy (16/19, 84%). The median duration of follow-up for all patients was 4.6 years (IQR, 2.8–7.7 years). Both the EP and Non-EP groups experienced frequent exacerbations of shadows on chest X-ray or CT; and in all patients in both groups, the appearance at recurrence was similar to that seen on previous CT (Table 2).

**Table 2:** Comparison of characteristics and laboratory data at recurrence between the EP and Non-EP groups

Characteristic	EP group (n = 6)	Non-EP group (n = 19)	
Recurrence	4 (67%)	15 (78%)	ns
Therapy			
Oral corticosteroids	5 (83%)	13/19 (68%)	ns
Anti-fungal drugs	1 (17%)	16/19 (84%)	0.0055
Duration (months) before recurrence, median (IQR)	22.5 (20.5–22.9)	20.5 (13.8–34.8)	ns
Total follow-up period (years), median (IQR)	4.0 (2.6–7.7)	4.6 (3.1–8.3)	ns
Recurrence rate/y, median (IQR)	0.23 (0.10–0.41)	0.38 (0.14–0.67)	ns
Phenotype at recurrence	EP: 4/4 (100%)	Non-EP: 16/16 (100%)	0.0002

Data are expressed as median and interquartile range (IQR) and categorical data are presented as number and percentage

*ns* not significant

### Relationship between peripheral eosinophil count and *Aspergillus*-specific IgE in EP and Non-EP groups

**Fig. 2:**
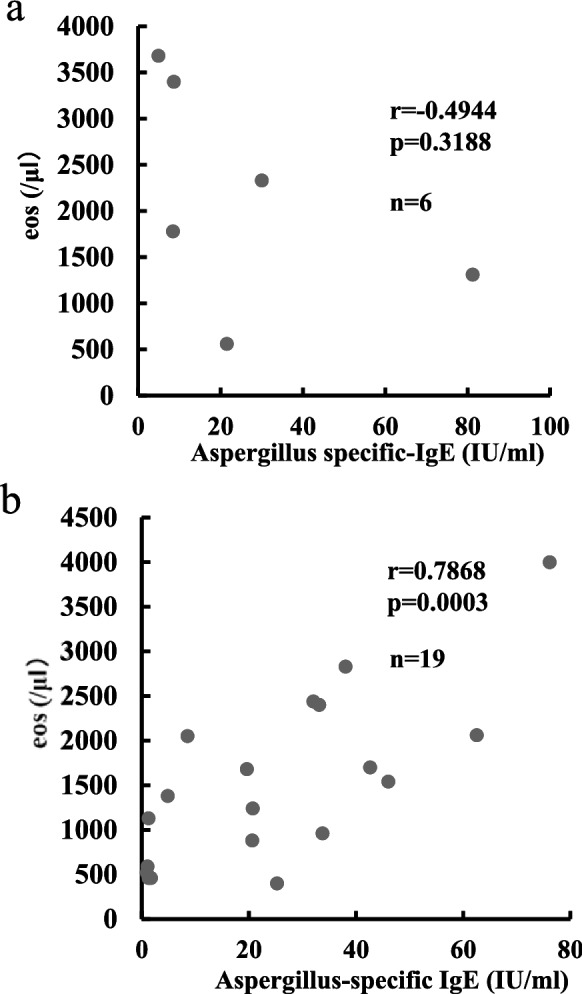
Relationship of peripheral eosinophil count and *Aspergillus*-specific IgE in the EP-group and Non-EP group. **a** EP group (n  = 6); **b** Non-EP group (n  = 19)

The relationships of peripheral eosinophils to *Aspergillus*-specific IgE were examined in both groups (Fig. 2). The EP group showed no significant correlation between the two (r  =  − 0.49, p  = 0.3188), possibly due to the small sample size, whereas the Non-EP group displayed a strong positive correlation (r  =  − 0.7878, p  = 0.0003). No significant correlation was seen between number of bronchial segments affected on HRCT and peripheral eosinophil count, total IgE, or *Aspergillus*-specific IgE in both groups (data not shown). Twelve patients in the Non-EP group showed HAM on HRCT, but no correlations were identified between the CT values of HAM and the blood data (eosinophil count, IgE, or *Aspergillus*-specific IgE) (data not shown).

### Results of serial CT in two cases in the EP group who received antifungal treatment

**Fig. 3:**
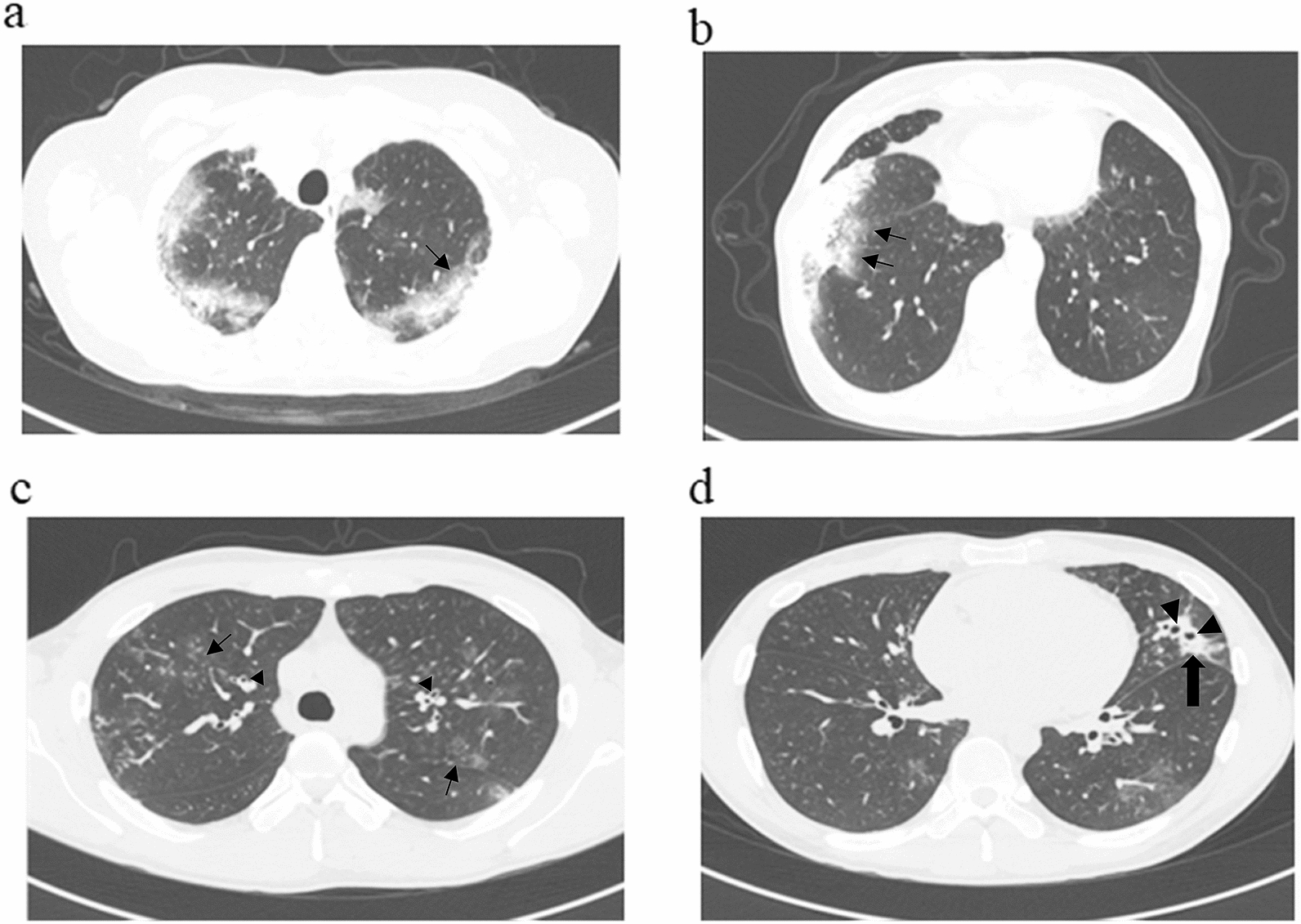
Representative HRCT images of the clinical course of patients in the EP group. **a** Case 1 at onset. HRCT shows ground-glass opacities in the peripheral upper lobes (arrow). **b** Case 1 at recurrence. HRCT demonstrates ground-glass opacities in the peripheral lower lobe (arrows). **c** Case 4 at the upper lobe level demonstrates ground-glass opacities (arrows), tree-in-bud appearance, and bronchial wall thickening (arrowheads). **d** Case 4 at the inferior lingular segment level shows central bronchiectasis (arrowheads) and peripheral consolidation (arrow)

Here, we describe two representative cases illustrating the typical course and HRCT findings in the EP group (Fig. 3).

#### Case 1

A 60-year-old woman with a history of infantile asthma. Her asthma control had been poor from the age of 57 years, and she experienced exacerbations needing oral corticosteroids about 5 times a year. In 2012, she was admitted to our hospital for further examination of eosinophilia (3680/μl) and abnormal shadows on chest X-ray. HRCT of the chest revealed bilateral peripheral-dominant infiltrative shadows with GGO, predominantly in the upper lobes (Fig. 3a). We administered systemic corticosteroid (prednisolone, 30 mg/day) under a presumptive diagnosis of eosinophilic pneumonia, and the abnormal shadows promptly disappeared. After treatment with oral corticosteroid (OCS), the diagnosis was changed to ABPA based on a positive reaction for *Aspergillu*s-specific IgE (4.9 UA/ml), *Aspergillus*-specific antibodies evaluated by precipitating antibodies, and fulfillment of the ISHAM criteria. Two years later, abnormal shadows in the right lower lobe appeared after she injured herself in a fall (Fig. 3b). We initially treated her with OCS (prednisolone, 20 mg/day), but diabetic control worsened, and her therapy was therefore switched to itraconazole. Antifungal treatment proved effective and mean morning peak flow increased from 280 L/min to 320 L/min, which was her personal best.

#### Case 2

A 25-year-old man with atopic dermatitis. In 2016, the patient was admitted to our hospital with a productive cough that had appeared 2 months prior. He had been diagnosed with BA by a previous doctor and had received OCS for 1 week, but symptoms had continued and laboratory data showed marked eosinophilia. HRCT showed mild central bronchiectasis, tree-in-bud sign, GGO, and p-consolidation (Fig. 3c). *A. niger* was detected in cultures of sputum and bronchial wash from left B5, and eosinophils were elevated to 61% in bronchial alveolar lavage fluid from left B5. Based on these data and positive specific IgE to *A. fumigatus*, ABPA was diagnosed and treatment with OCS (0.5 mg/kg/day) and itraconazole (200 mg/day) was initiated. His symptoms and the abnormal shadows on chest XP improved steadily.

## Discussion

In this study, we demonstrated a subtype of ABPA that shows similar imaging findings to CEP, which we investigated as the EP group. The EP group showed three major characteristics, as follows. First, HRCT showed peripheral-dominant consolidation and GGO but no HAM in the central airways. Second, most cases did not demonstrate fungal infection in cultures of sputum or bronchial wash, and beta-D glucan levels were not elevated. Third, *Aspergillus*-specific IgE was elevated in all cases and *Aspergillus-*specific precipitating antibody or IgG was also positive in all cases. In contrast, patients in the Non-EP group, who had classical-type ABPA, showed mucoid impaction and HAM on HRCT indicating a relationship with *Aspergillus* infection, related to the positive correlation between peripheral eosinophil count and *Aspergillus*-specific IgE. Recurrence was common in both groups. Of note, the main characteristics of HRCT at onset were unchanged at recurrence, in both groups.

Several studies have reported a relationship between HRCT findings and immunologic makers and prognosis in patients with ABPA. ABPA-S was proposed by Patterson et al*.* in 1986, and defined as filling the diagnostic criteria for ABPA but without evidence of proximal bronchiectasis, and ABPA-CB was with proximal bronchiectasis [17]. ABPA-S is reported to show a tendency of lower concentrations of total IgE and *Aspergillus*-specific IgE [18], and have good outcome in terms of no progression to ABPA-CB and maintenance of pulmonary function [19]. However, one-third or more of patients with ABPA-S have recurrence during the course and oral corticosteroid treatment is required [19]. In the present study, both groups experienced relapse with approximately the same frequency, and it was confirmed that the patient groupings remained unchanged even after recurrence. From this perspective, the relationship between the present two groups resembles the linkage between ABPA-S and ABPA-CB. However, the EP group did not show lower concentrations of serum IgE and anti-*Aspergillus*-specific IgE than the Non-EP group; and as mentioned above, the groupings remained unchanged following disease progression. Therefore, the EP group is not a serologically mild form or an early stage of ABPA, but another subclassification of ABPA that is different from ABPA-S.

Moreover, a diabetic patient in the present EP group was treated effectively with an antifungal compound. According to Agarwal et al. itraconazole and voriconazole have shown equivalent therapeutic effects for ABPA as standard corticosteroid therapy [20, 21], which indicates that antifungal treatment is an appropriate choice for patients in the EP group.

Another study has stated that ABPA is the biggest cause of pulmonary eosinophilia in Britain [8] and reported that episodes of pulmonary eosinophilia are more frequent in patients with ABPA than in those with pulmonary eosinophilia without aspergillosis. In the present study, 50% of the EP group and 26% of the Non-EP group had a history of CEP. Among these, several patients might have been in the early phase of ABPA. As reported in the Results, none of the three patients in the EP group had been checked for *Aspergillus*-specific IgE (a required item in the ISHAM criteria) at the time of diagnosis of CEP. Furthermore, in three patients in the Non-EP group, slight HAM on HRCT in the past CEP phase had been overlooked in our retrospective review. In a previous study regarding the diagnosis of ABPA, the sensitivity of *A. fumigatus*-specific IgE level  > 0.0.35 IU/ml was reported as 100% and the specificity of HAM was reported as 100% [22]. Accordingly, if the present patients with a history of CEP had been diagnosed with ABPA earlier, recurrence could have been prevented. Furthermore, of the present patients, five progressed from BA to ABPA within 3 months, among whom three showed only continuous cough without typical wheezing as the initial symptom. Taking such findings into consideration, ABPA must be considered in the differential diagnosis when we treat patients with eosinophilia and pulmonary infiltrative shadows, even without a history of asthma. In other words, we should also check for *Aspergillus*-specific IgE and for the presence or absence of HAM on HRCT in patients with pulmonary eosinophilia.

A possible problem is how the onset of ABPA can be distinguished from the clinical course of CEP, as described above. Two previous case reports have described CEP progressing to ABPA [10, 11]. However, no reports have focused on the possibility that CEP-like shadows on chest CT could be primary lesions of ABPA. Asano et al*.* recently discussed new clinical diagnostic criteria for ABPA/allergic bronchopulmonary mycosis (ABPM), using CEP as a control population [9]. They reported that 50% of cases of CEP exhibited asthma and that several of these (4.7%) were compatible with ABPA/ABPM, based on the ISHAM criteria and the newly proposed criteria for ABPM. In the present study, 5 out of 6 patients in the EP group matched both of these criteria, and all of the Non-EP group met the ABPM criteria. The criteria proposed by Asano et al*.* comprised 10 components and placed great importance on the presence of fungi in the mucus and on CT change in the central bronchus due to mucus plugs. Patients in the present EP group fulfilled the ISHAM diagnostic criteria, which emphasizes sensitization for *Aspergillus* and not necessarily the presence of change in the central bronchus. Therefore, patients in the EP group did not always adapt to the newly proposed criteria.

In our study, the EP group showed a non-significant tendency toward a negative correlation between peripheral eosinophils and *Aspergillus*-specific IgE. In contrast, the Non-EP group showed a clear positive correlation between these two values. In ABPA, peripheral eosinophil count has been reported to be much higher in cases with HAM than in those without HAM, and *Aspergillus*-specific IgE tends to be increased in cases with HAM [23, 24]. However, no reports have described such a positive correlation between peripheral eosinophils and *Aspergillus*-specific IgE in ABPA patients. The present results indicate differences in the mechanism of pathogenesis between groups and the implications of dividing ABPA into these two groups. Furthermore, in our study, beta-D glucan was higher in the Non-EP group than in the EP group and peripheral eosinophilia was prominent in the EP group. These results suggest a closer relationship with T-helper 2 (Th2) inflammation from fungal airway infection in the Non-EP group than in the EP group, whereas the EP group might have a closer correlation with eosinophilic inflammation induced by type 2 innate lymphoid cells (ILC2s), as has been previously described in patients with severe asthma and fungal sensitization [25].

Limitations of this study include the small number of eligible patients and the single-center retrospective design. However, the baseline characteristics of our study population corresponded to those of cases in a nationwide survey of ABPA in Japan by Oguma et al*.* [6], in which the peripheral blood data (e.g., eosinophil count and IgE level) were similar to those in the present results. Therefore, we consider that the present results have profound significance for the accurate diagnosis of ABPA and for the selection of effective therapy.

In conclusion, we have identified a subgroup of ABPA in which the imaging findings resemble those of CEP but the clinical course differs from typical ABPA. When we encounter pulmonary eosinophilia, *Aspergillus*-specific IgE should be checked, with awareness of the existence of non-typical ABPA.

## Data Availability

The data collected and analyzed during the current study are available from the corresponding author on reasonable request.

## Abbreviations

ABPA: Allergic bronchopulmonary aspergillosis
ABPA-S: Serologic ABPA
ABPM: Allergic bronchopulmonary mycosis
BA: Bronchial asthma
CEP: Chronic eosinophilic pneumonia
EP: Eosinophilic pneumonia
GGO: Ground-glass opacities
Ig: Immunoglobulin
ISHAM: International Society of Human and Animal Mycology
HAM: High-attenuation mucus
HRCT: High-resolution CT
NTM: Non-tuberculous mycobacteria
p-consolidation: Peripheral consolidation
TB: Tuberculosis

## References

[1] Agarwal R (2009). Allergic bronchopulmonary aspergillosis. Chest.

[2] Agarwal R, Chakrabarti A, Shah A, Gupta D, Meis JF, Guleria R, Moss R, Denning DW (2013). Allergic bronchopulmonary aspergillosis: review of literature and proposal of new diagnostic and classification criteria. Clin Exp Allergy.

[3] Smits HH, Hiemstra PS, da Costa PC, Ege M, Edwards M, Garn H, Howarth PH, Jartti T, de Jong EC, Maizels RM, Marsland BJ, McSorley HJ, Müller A, Pfefferle PI, Savelkoul H, Schwarze J, Unger WW, von Mutius E, Yazdanbakhsh M, Taube C (2016). Microbes and asthma: opportunities for intervention. J Allergy Clin Immunol.

[4] Rosenberg M, Patterson R, Mintzer R, Cooper BJ, Roberts M, Harris KE (1977). Clinical and immunologic criteria for the diagnosis of allergic bronchopulmonary aspergillosis. Ann Intern Med.

[5] Glancy JJ, Elder JL, McAleer R (1981). Allergic bronchopulmonary fungal disease without clinical asthma. Thorax.

[6] Oguma T, Taniguchi M, Shimoda T, Kamei K, Matsuse H, Hebisawa A, Takayanagi N, Konno S, Fukunaga K, Harada K, Tanaka J, Tomomatsu K, Asano K (2018). Allergic bronchopulmonary aspergillosis in Japan: a nationwide survey. Allergol Int.

[7] Agarwal R, Gupta D, Aggarwal AN, Saxena AK, Chakrabarti A, Jindal SK (2007). Clinical significance of hyperattenuating mucoid impaction in allergic bronchopulmonary aspergillosis: an analysis of 155 patients. Chest.

[8] Chapman BJ, Capewell S, Gibson R, Greening AP, Crompton GK (1989). Pulmonary eosinophilia with and without allergic bronchopulmonary aspergillosis. Thorax.

[9] Asano K, Hebisawa A, Ishiguro T, Takayanagi N, Nakamura Y, Suzuki J, Okada N, Tanaka J, Fukutomi Y, Ueki S, Fukunaga K, Konno S, Matsuse H, Kamei K, Taniguchi M, Shimoda T, Oguma T (2021). New clinical diagnostic criteria for allergic bronchopulmonary aspergillosis/mycosis and its validation. J Allergy Clin Immunol.

[10] Lee JY, Choi H, Chon GR (2014). Allergic bronchopulmonary aspergillosis mimicking relapsing chronic eosinophilic pneumonia in non-asthma patient. Allergol Int.

[11] Mochimaru T, Fukunaga K, Ueda S, Kuroda A, Watanabe R, Chubachi S, Betsuyaku T (2019). Allergic bronchopulmonary aspergillosis in succession to chronic eosinophilic pneumonia. Allergol Int.

[12] Global Strategy for Asthma Management and Prevention 2019 update. https://ginasthma.org/wp-content/uploads/2019/06/GINA-2019-main-report-June-2019-wms.pdf. Accessed 29 Oct 2020.

[13] Hansell DM, Strickland B (1989). High-resolution computed tomography in pulmonary cystic fibrosis. Br J Radiol.

[14] Logan PM, Müller NL (1996). High-attenuation mucous plugging in allergic bronchopulmonary aspergillosis. Can Assoc Radiol J.

[15] Cottin V (2016). Eosinophilic lung diseases. Clin Chest Med.

[16] Suzuki Y, Suda T (2019). Eosinophilic pneumonia: a review of the previous literature, causes, diagnosis, and management. Allergol Int.

[17] Patterson R, Greenberger PA, Halwig JM, Liotta JL, Roberts M (1986). Allergic bronchopulmonary aspergillosis. Natural history and classification of early disease by serologic and roentgenographic studies. Arch Intern Med.

[18] Greenberger PA, Miller TP, Roberts M, Smith LL (1993). Allergic bronchopulmonary aspergillosis in patients with and without evidence of bronchiectasis. Ann Allergy.

[19] Agarwal R, Garg M, Aggarwal AN, Saikia B, Gupta D, Chakrabarti A (2012). Serologic allergic bronchopulmonary aspergillosis (ABPA-S): long-term outcomes. Respir Med.

[20] Agarwal R, Dhooria S, Sehgal IS, Aggarwal AN, Garg M, Saikia B, Chakrabarti A (2018). A randomised trial of voriconazole and prednisolone monotherapy in acute-stage allergic bronchopulmonary aspergillosis complicating asthma. Eur Respir J.

[21] Agarwal R, Dhooria S, Singh Sehgal I, Aggarwal AN, Garg M, Saikia B, Behera D, Chakrabarti A (2018). A randomized trial of itraconazole vs prednisolone in acute-stage allergic bronchopulmonary aspergillosis complicating asthma. Chest.

[22] Agarwal R, Maskey D, Aggarwal AN, Saikia B, Garg M, Gupta D, Chakrabarti A (2013). Diagnostic performance of various tests and criteria employed in allergic bronchopulmonary aspergillosis: a latent class analysis. PLoS ONE.

[23] Agarwal R, Khan A, Gupta D, Aggarwal AN, Saxena AK, Chakrabarti A (2010). An alternate method of classifying allergic bronchopulmonary aspergillosis based on high-attenuation mucus. PLoS ONE.

[24] Phuyal S, Garg MK, Agarwal R, Gupta P, Chakrabarti A, Sandhu MS, Khandelwal N (2016). High-attenuation mucus impaction in patients with allergic bronchopulmonary aspergillosis: objective criteria on high-resolution computed tomography and correlation with serologic parameters. Curr Probl Diagn Radiol.

[25] Castanhinha S, Sherburn R, Walker S, Gupta A, Bossley CJ, Buckley J, Ullmann N, Grychtol R, Campbell G, Maglione M, Koo S, Fleming L, Gregory L, Snelgrove RJ, Bush A, Lloyd CM, Saglani S (2015). Pediatric severe asthma with fungal sensitization is mediated by steroid-resistant IL-33. J Allergy Clin Immunol.

